# A novel library of -arylketones as potential inhibitors of α-glucosidase: Their design, synthesis, *in vitro* and *in vivo* studies

**DOI:** 10.1038/s41598-017-13798-y

**Published:** 2017-10-16

**Authors:** Tania Luthra, Rahul Agarwal, Mamidala Estari, Uma Adepally, Subhabrata Sen

**Affiliations:** 1 Department of Chemistry, School of Natural Sciences, Shiv Nadar University, Dadri, Chithera, GautamBudh Nagar, Uttar Pradesh 201314 India; 2 Institute of Science and Technology Jawaharlal Nehru Technological University, Kukatpally, Hyderabad, Telangana India; 3 Department of Life Sciences, School of Natural Sciences, Shiv Nadar University, Dadri, Chithera, GautamBudh Nagar, Uttar Pradesh 201314 India; 4 Department of Zoology, Kakatiya University, Warangal-506009, Telengana, India

## Abstract

α-glucosidase is an essential enzyme located at the brush border of intestines. It is an important therapeutic target for type II diabetes. Herein we have designed a library of novel α-arylketones as inhibitors of α-glucosidase (yeast origin) *via* scaffold hopping and bioisosteric modification of known inhibitors of α-glucosidase. The design was validated through molecular docking that revealed strong binding interactions of the newly designed compounds against α-glucosidase. A library comprising of 15 compounds was synthesized in a combinatorial fashion, where the advanced amide intermediates were accessed through “shot gun” synthesis. The final compounds were characterized by ^1^H, ^13^C-NMR and with high resolution mass spectroscopy. *In vitro* screening of the compounds against yeast α-glucosidase revealed substantial inhibition with IC_50_s in the range of 4–10 μM (the standard drug acarbose inhibits α-glucosidase with an IC_50_ of 9.95 μM). Reaction kinetics suggested mixed type inhibition. Finally, *in vivo* studies of the most active compound **3c** against Streptozotocin induced male albino Wistar rats revealed that its administration in the rats for about 4 weeks lead to a highly significant (P < 0.001) decrease in the fasting blood glucose (FBG) compared to the untreated diabetic rats. Moreover, lower dose of **3c** had better control over FBG in contrast to high-dose.

## Introduction

α-glucosidase is an exocyclic enzyme that hydrolyses the 1,4-α-glycosidic linkages of oligosaccharides. The oligosaccharides are then converted to monosaccharides which are then absorbed into the blood from the intestine^1^. Blood carries these monosaccharides to the individual cells where they are converted into energy by the cell machinery with the help of insulin. At times the cells fail to recognize insulin or insufficient amount of insulin gets secreted from the pancreas. Consequently the monosaccharides failed to get converted to the energy and are retained in the blood, which in turn increases the blood glucose level, leading to hyperglycemia. This condition leads to type II diabetes one of the most fatal non communicable disease of the modern world^2^. Hence α-glucosidase is an essential target for type II diabetes and their inhibitors are used to alleviate the disease^3^. These inhibitors bind to α-glucosidase and delay the absorption of carbohydrates from the small intestine, thereby lowering the postprandial blood glucose level^4^. In last few years various small molecules have been reported as potential inhibitors of α-glucosidase^5–9^. For example, 5-bromo-2-arylbenzimidazole derivatives exhibited dual inhibition against α-glucodiase and ureas enzymes. Additionally they are non-cytotoxic by nature^10^. In another report molecular modeling studies generated 3-thiazolyl coumarine derivatives as effective inhibitors against α-glucosidase, with IC_50_ in the range of 0.12–16 μM^11^. A one pot multicomponent reaction involving aryl hydrazide/phenyl hydrazine and benzophenone with appropriate aryl thiocyanates and phenacyl bromide afforded novel thiazoles which inhibit α-glucosidase with IC_50_ in the range of 9–82 μM^12^. Apart from small molecules natural product extracts have also demonstrated inhibition of α-glucosidase enzyme^13^.

Nojirimycin, Miglitol, Voglibose and Acarbose are marketed drugs for type II diabetes which target α-glucosidase enzyme^14–17^. In general α-glucosidase inhibitors are advantageous over other antidiabetic drugs due to their localized action and minimum absorption (this subsequently reduces the systemic side effects)^18^. However the aforementioned drugs are quite old (discovered about 30–40 years) and suffer from disadvantages related to flatulence, abdominal pain and diarrhea due to microbial action on undigested carbohydrates^19^. Hence development of alternative α-glucosidase inhibitor is always a promising drug discovery endeavor. Herein we report, design of a series of α-arylketones as novel α-glucosidase inhibitors through scaffold hopping and bioisosteric modification of known α-glucosidase inhibitors. The design was rationalized through molecular docking. The compounds were synthesized in a parallel fashion, where the advanced intermediates were synthesized *via* “shot gun” synthesis^20^. Finally the compounds were subjected to *in vitro* screening against yeast α-glucosidase and *in vivo* screening in Streptozotocin induced male albino wistar rats, to assess their potential as candidates against type II diabetes.

Scaffold hopping is a technique where the central core of a bioactive molecule is replaced with an appropriate chemical motif, in a bid to generate a new molecule with equal or better potency compared to the original molecule^21^. This is also done to augment the selectivity (against the relevant target) and ADMET properties. For example Merck identified a piperidine based T-type Ca^2+^ inhibitors, which were later scaffold hopped to a new motif containing pyrrolidine fused with cyclopropane ring system. These newer compounds were favorable lead molecules over the piperidine class of molecules, exhibiting much better *in vivo* results^22^.

Bioisosteric modification is another lead optimization technique where, certain substituents in the original molecule are replaced with suitable functionalities that can potentially improve physicochemical parameters such as ADMET, pk/pd, toxicity and etc. of the original molecule^23^. For example, in a bid to discover anti-inflammatory drugs acting through the selective inhibition of prostaglandin-H synthase-2 (PGHS-2) or cyclooxygenase-2 (COX-2) the scientists from Pfizer/Searle discovered a pyrazole based compound SC-58125. Despite good selectivity profile and excellent potency, this drug reported a half-life of ~200 h, thereby revealing poor susceptibility during its metabolism in the liver. Bioisosteric replacement of the methyl and the fluorine functionality in the molecule with –NH_2_ and –CH_3_ reduced the half-life to 8–12 h, thereby making it compatible for introducing it in the market as an anti-inflammatory agent^24^.

Finally “shot gun” synthesis is a procedure where multiple products are generated simultaneously in a reaction. This can happen when individual reactions occur at rates sufficiently different to allow them to progress in a same reaction chamber without interference^25^.

## Results and Discussions

### Design

**Figure 1:**
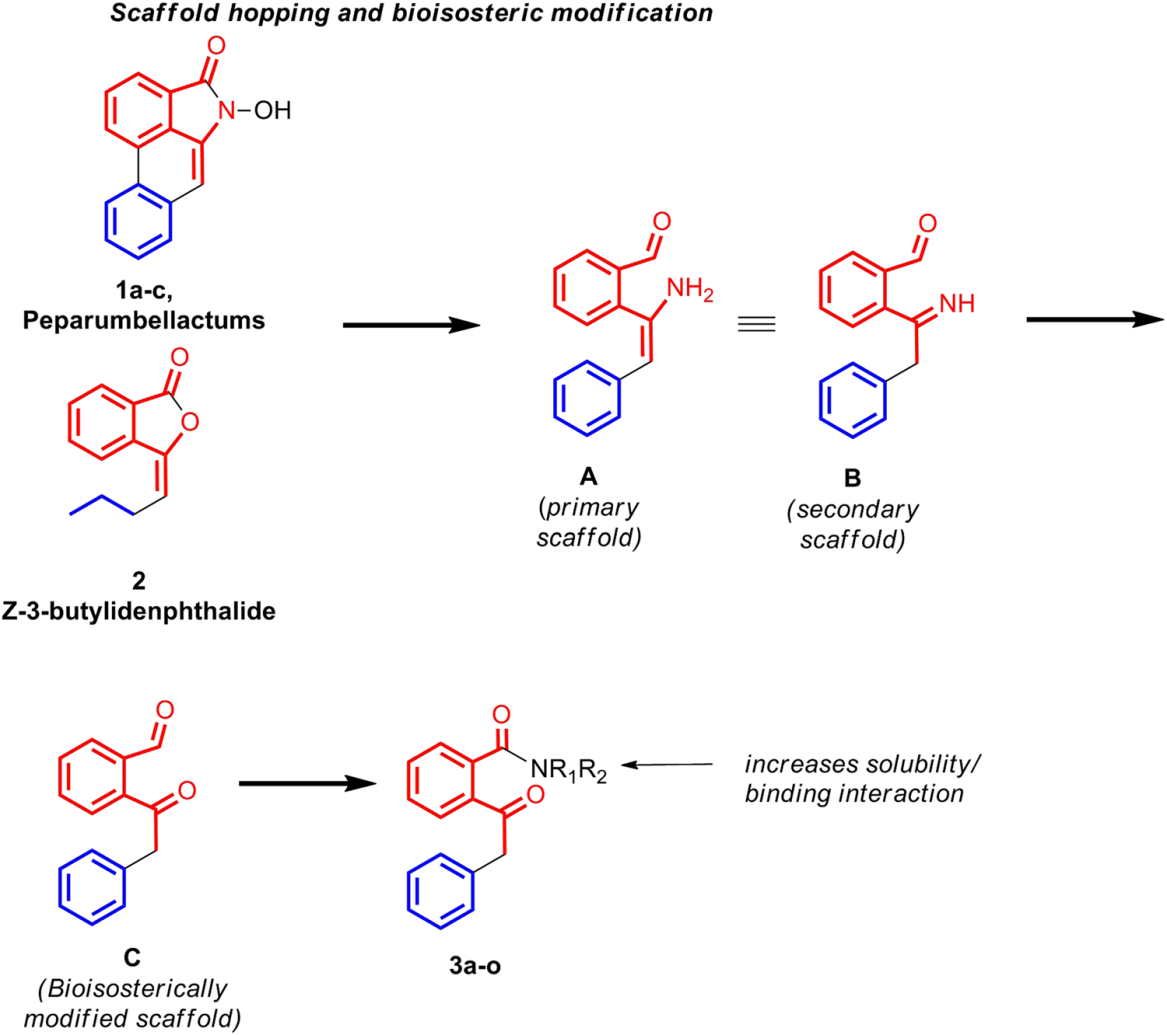
Design of novel α-glucosidase inhibitors by scaffold hoping and bioisosteric modification.

In a bid to desi gn novel inhibitors against α-glucosidase, we interrogated the structural features of plethora of small molecules reported in the literatures as inhibitors of α-glucosidase. We selected few molecules with moderate to good activity, which would provide us with the scope of further improving the activity through our newly designed molecule. Accordingly compounds **1a**-**c** and **2** were selected^26,27^. **1a**-**c** are alkaloids called *Peparum Bellactams*, isolated from the branches of *Piper Umbellatum*. They exhibited moderate inhibitory activity against α-glucosidase (yeast) with IC_50_ of 30–90 μM (Fig. 1)^26^. Compound **2**, lowered blood glucose level in NA-STZ-diabetic mice at 10, 31.2 and 56.2 mg/Kg (Fig. 1)^27^. A closure look at these two classes of molecules revealed topological similarity between them as depicted by fragment **A** in Scheme 1. The stilbene moiety is incorporated from **1a**-**c**. Bioisosteric shuffling of the imine nitrogen in **B** with oxygen resulted in scaffold **C**. Final scaffold modification involved installation of the amide functionality (inspired from **1a**-**c**) to generate the desired molecules **3a**-**o**.

**Figure 2:**
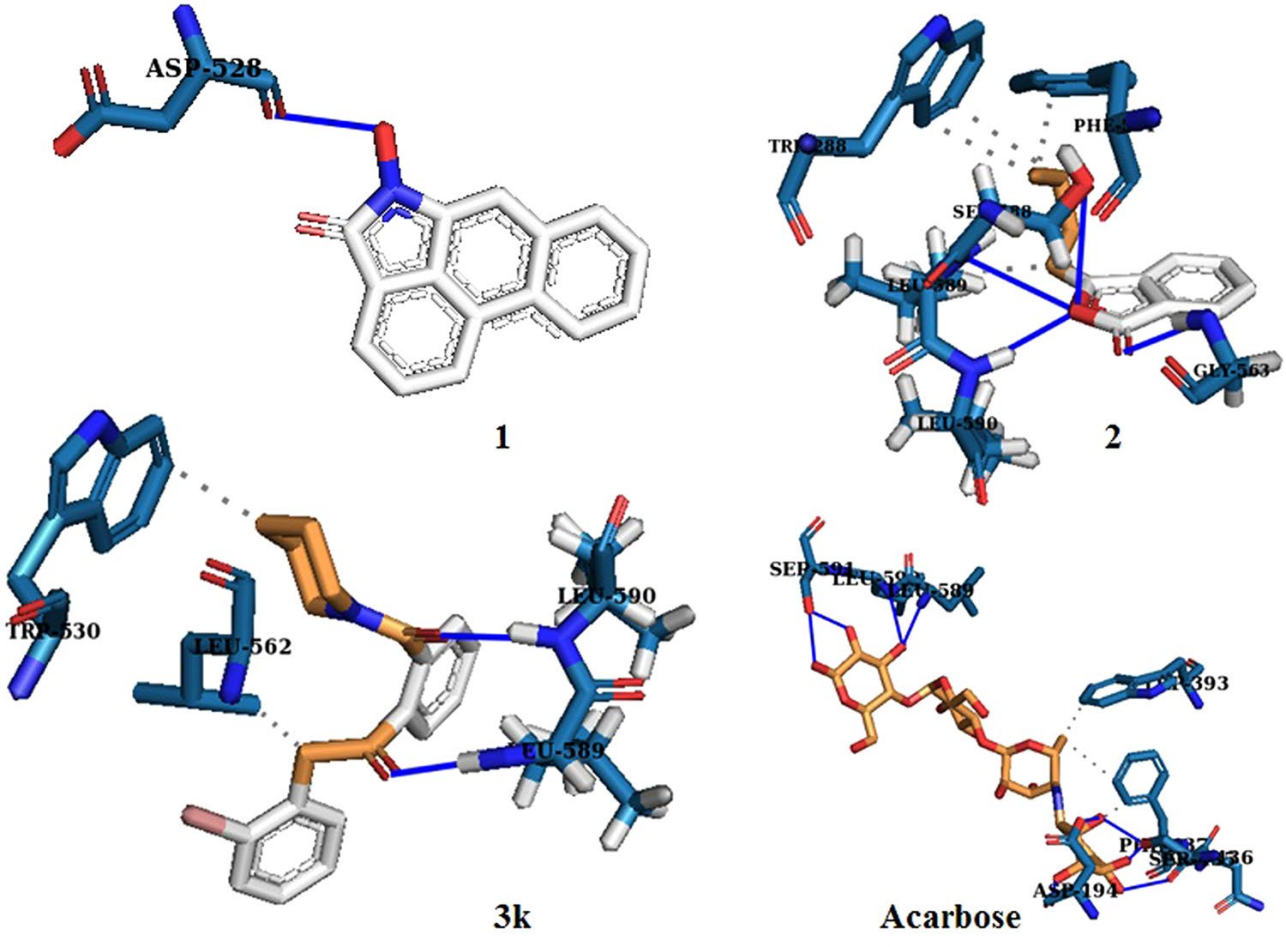
Molecular docking of **1**, **2**, **3k** and **acarbose** with a homology model of α-glucosidase.

Next the design of our new molecular scaffolds were rationalized by comparing the binding interaction of **3k** (a representative molecule of our library) and a homology model of α-glucosidase along with **1**, **2** and acarbose (a standard drug) (Fig. 2). The homology model was created from human maltase glucoamylase (PDBID: 2QLY, 3L4T) which exhibited strong similarity with the query sequence. The stereochemical quality of this model was validated by Ramachandran plot which revealed that ~99.3% of residue are in favored and allowed regions with only 0.7% outliers (refer *SI*).

From the docking interactions it was observed that compound **3k**, could potentially bind more strongly to α-glucosidase than **1** and **2** (Fig. 2). Furthermore, close scrutiny revealed comparable hydrogen bonding and hydrophobic interactions between the original molecules (**1** and **2**) and **3k** with α-glucosidase (Fig. 2). The predicted binding conformations of **1** indicate one hydrogen bond interaction with the α-glucosidase protein model where the hydroxyl group of **1** bonds with the carboxylate group of the Asp528 residues having a distance of 1.80 Å. Compound **2** indicate four hydrogen bonding interactions with residues Gly563, Ser588, Leu589 and Leu590 (Fig. 2). The three hydrogen bonds were formed between the amino group (N-H) of Ser588, Leu589, Leu 590 residues and the ring oxygen of the lactone ring with a distance of 3.44 Å, 3.04 Å and 2.14 Å respectively. The fourth hydrogen bond was observed between the amino group (N-H) of Gly563 residues and the carbonyl oxygen of the lactone moiety in **2** with a distance of 2.37 Å. Compound **2** exhibits hydrophobic interaction with the Trp288, Phe561 and Leu589 residues (Fig. 2). The binding interactions of compound **3k** involve two hydrogen bonds in which the carbonyl oxygens of compound **3k** are bonded with the amino group (N-H) of Leu589 and Leu590 respectively with a distance of 2.14 Å and 1.72 Å. Compound **3k** also displayed hydrophobic interaction with the residues Trp530 and Leu562 (Fig. 2). Finally, acarbose interacted with the homology model, through six amino acid residues *via* nine hydrogen bonds and two amino acids *via* hydrophobic interaction. The hydrophobic interactions were observed with Trp393 and Phe437. The hydrogen bonds were present between ring hydroxy groups of acarbose and the carboxylate functionality of Asp194, Ser435, Asn436, Leu589, Leu590, Ser591 residues. The subsequent docking scores are −6.11, −5.33, −7.21 and −4.45 kcal/mol for **1**, **2**, **3k** and acarbose respectively.

### Chemistry

**Figure 3:**
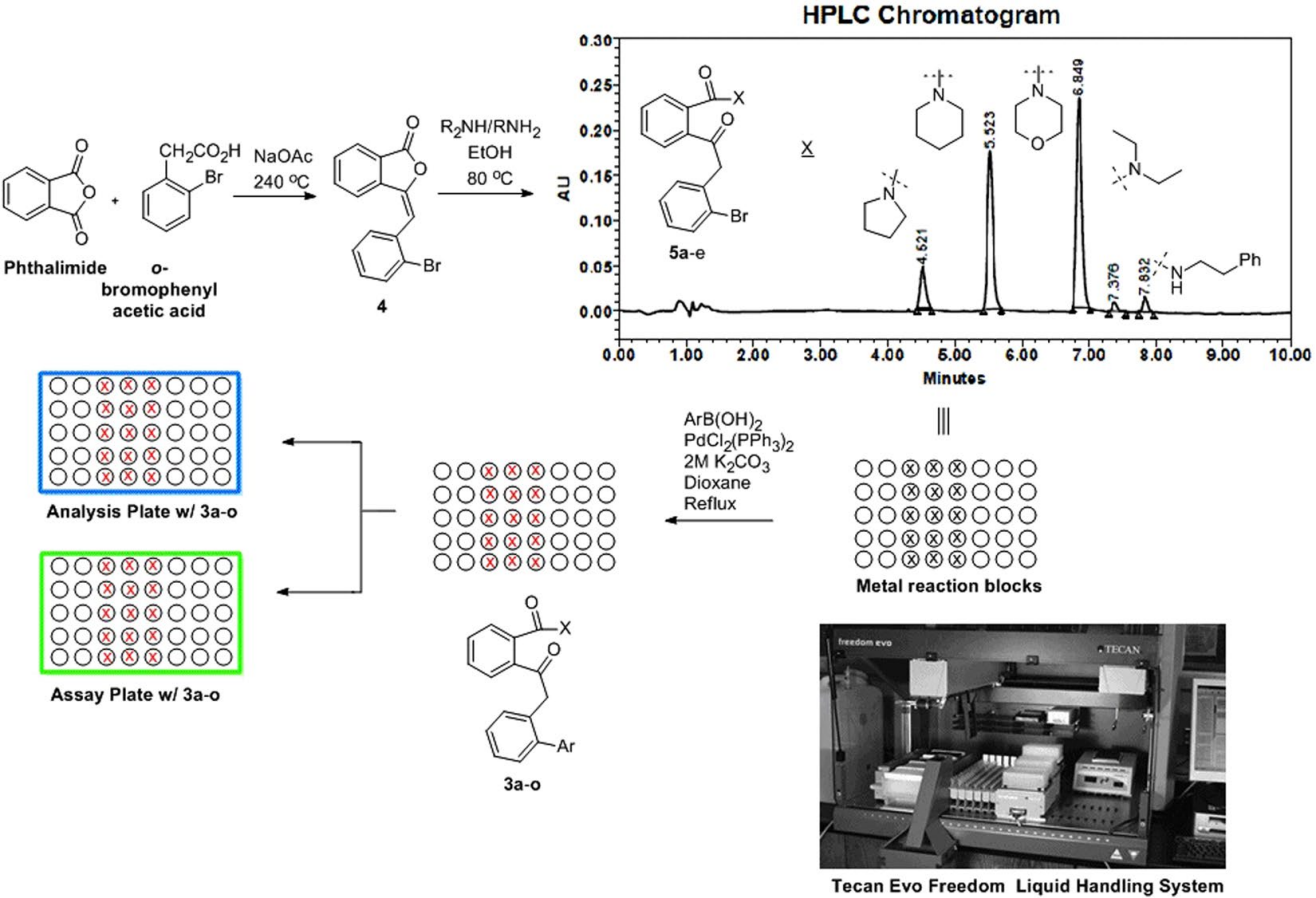
Synthesis of the library of compounds **3a**-**o**.

Based on the docking results we envisioned a focused library of 15–20 members based on compound **3k**. Accordingly Knoevenagel condensation of pthalimide with *o*-bromophenyl acetic acid in sodium acetate (NaOAc) afforded compound **4** (Fig. 3). This was subjected to a “shot gun” synthesis to obtain a series of next five intermediates **3k**-**o** ^28,29^. A typical procedure involved dissolving five equivalents of **4** in morpholine, piperidine, pyrrolidine, diethyl amine andphenethyl amine (one equivalent each) and subsequent stirring at room temperature. The reaction was monitored by HPLC (high performance liquid chromatography) and when it indicated completion of **4** (Fig. 3), the reaction was quenched with water to remove any excess unreacted amine and was extracted with ethyl acetate. The organic layer was dried over anhydrous magnesium sulphate, evaporated and the crude was subjected to flash column chromatography (twice) for the isolation of the intermediate amides **3k**-**o** (Fig. 3). The final Suzuki reactions between the amides **3k**-**o** with appropriate boronic acids were conducted in parallel within custom made metallic reaction blocks in pre-tared reaction vessels containing 0.05 molar solution of amides **3k**-**o** (scale: 50–100 mg) in dioxane with palladium chloride bistriphenyl phosphine (PdCl_2_(PPh_3_)_2_) and aqueous potassium carbonate (K_2_CO_3_) (Fig. 3). The reactions were monitored by LCMS and once it indicates complete consumption of the starting amides, the reactions were quenched and the products were extracted with ethyl acetate in a shaker. The supernatant ethyl acetate layers were transferred to a different set of pre-tared vials, evaporated under high vacuum and the net amount of compounds were deduced (Fig. 3). 2 mg of samples (including that of **3k**-**o**) were transferred to a 96 well analytical plate, which was subjected to HPLC analysis to assess the purity of the products. To our utmost gratification eight out of the ten compounds of **3a**-**j** exhibited > 95% purity. The remaining two were washed multiple times with hexane to achieve the desired purity. An assay plate was also generated containing 50 μM solution of compounds in dimethyl sulphoxide. The whole operation was executed through a TecanEvo Freedom liquid handling system containing robotic arms (Fig. 3). The average yield of the analogs ranged from 79–94%. All the compounds were further characterized by ^1^H,^13^C-NMR and high resolution mass spectroscopy.

### *In vitro* screening and structure activity relationship studies

To assess the inhibitory effect and also the extent of selectivity our library was against α-glucosidase (yeast origin), α-fucosidase (bovine kidney origin) and α-mannosidase (jack bean origin) (in 96-well plates employing 4-nitrophenyl-α-D-glucopyranoside (PNPG) as substrate) (Table 1). The test compounds were dissolved and diluted in dimethyl sulfoxide (DMSO). They were placed in the plate along with the substrate and 10 mM of potassium sulphate buffer. They were then reacted with appropriate enzymes. After incubating the plates for ten minutes, the absorbance of the wells were measured at 430 nM. The increase in abundance (ΔA) was compared against the control i.e. buffer. The inhibitory concentrations (IC_50_s) were computed using the standard formula (Inhibition% = ΔA_control_ − [ΔA_sample_/ΔA_control_] × 100).Acarbose, is used as a standard drug for this assay (Table 1).

**Table 1:** Screening of compounds **3a**-**o** against variety of α-glycosidases.

Compounds (structures)	Compound #s	IC_50_s in μM^a^		
		α-glucosidase (yeast)	α-fucosidase (bovine kidney)	α-mannosidase (jack bean)
	3a	5.93	>50	>50
	3b	5.88	>50	>50
	3c	4.50	>50	>50
	3d	5.68	>50	>50
	3e	4.93	34.43	>50
	3 f	5.95	48.08	>50
	3 g	5.15	>50	49.41
	3 h	5.17	>50	>50
	3i	5.93	>50	23.82
	3j	5.89	>50	44.78
	3k	5.81	>50	36.21
	3 l	5.20	>50	>50
	3 m	10.15	>50	>50
	3n	10.13	>50	>50
	3o	6.93	>50	>50
Acarbose	—	9.95		

^a^IC_50_was determined from two independent assays, performed in duplicate.

**Figure 4:**
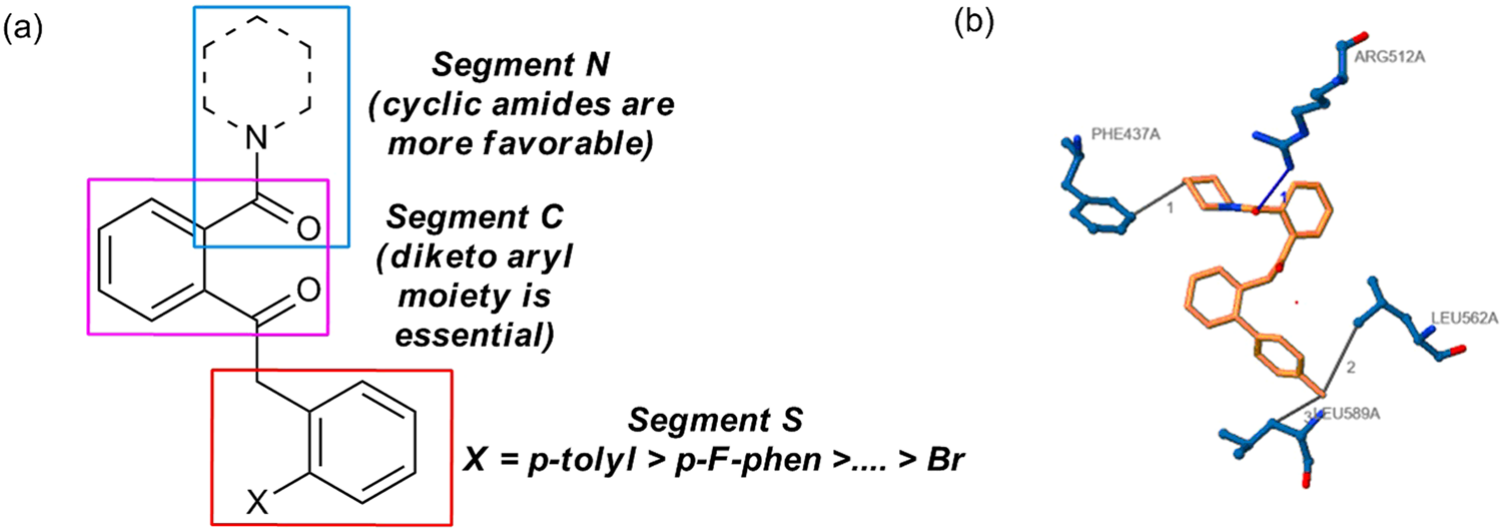
(**a**) SAR studies of our library against yeast α-glucosidase; (**b**) Binding interactions of **3c** with the homology model of α-glucosidase.

From *in vitro* screening it is evident that the structure activity relationship (SAR) in this series of molecules is extremely constricted. In general the molecules have shown decent inhibitory activity against yeast α-glucosidase with IC_50_ ranging from 4.5–10.2 μM. Interestingly, they have not exhibited any substantial inhibition in α-fucosidase (bovine kidney) or α-mannosidase (jack bean) indicating high selectivity of binding with α-glucosidase. The most active compound **3c** exhibited inhibition of 4.5 μM. To explain the SAR, the molecular architecture is divided into three segments. The northern most segment (N) consists of various amides, the central segment (C) is the diketoaryl moiety and finally the aromatic region in the south segment (S) (Fig. 4). From the initial SAR it is obvious that the centrally located diketoaryl moiety is essential to impart inhibition in yeast α-glucosidase. In segment N, variety of cyclic amines such as piperidine, pyrrolidine, and morpholine are well tolerated, as exemplified by low micromolar IC_50_s of **3a** - **l** (Table 1). However the phenethyl amine analog **3o** was least effective. A closure look at the aromatic region in the south segment (S), revealed that other than **3k** the *o*-bromo aromatics such as **3** **m** and **n** are not as potent as the other aromatic compounds such as **3c** - **e** (10.1 → 4.49 μM). It is noteworthy that our most active compound **3c**, was twice as active as the standard drug acarbose (IC_50_ 4.5 *Vs* 9.95 μM). From this initial SAR we conclude that pyrrolidine and piperidines are tolerated in segment N. The diketoaryl moiety at the center is essential and substituted (with methyl) aromatics down south (S) facilitates the activity. Furthermore, the docking study of **3c** with the homology model of α-glucosidase revealed several critical binding interactions (including one hydrogen bonding and three hydrophobic interactions) between them which explains its high inhibitory activity (Fig. 4).

### Reaction kinetics

**Figure 5:**
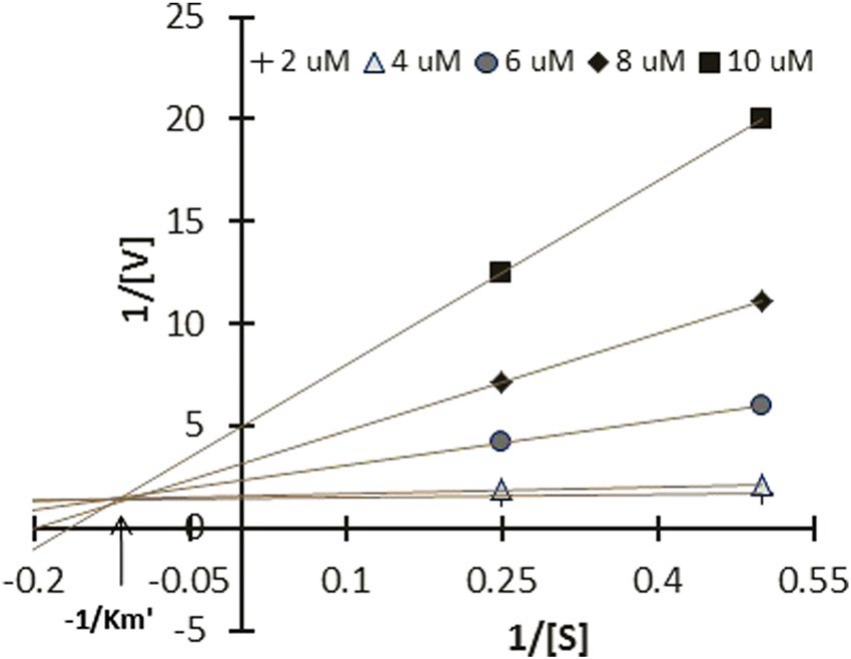
LB plot and secondary plot of **3c** and α-glucosidase (yeast origin) binding studies indicated a mixed type inhibition.

Inhibition kinetics assay of the most active compound **3c**, was performed to understand the mode of action of the compound against α-glucosidase (yeast origin). The inhibition kinetics was determined graphically by using primary (Lineweaver-Burk) and secondary plots. Figure 5, depicted the α-glucosidase inhibition kinetics plot for compound **3c** (refer *SI* for raw data). It revealed that all the data points on the Lineweaver-Burk plot of **3c** get intersected in the second quadrant, which indicated a mixed type of inhibition. This indicated binding of **3c** with the enzyme (α-glucosidase) as well as the enzyme-substrate (α-glucosidase-PNDP) complex. This generated two inhibition constants, K_i_ and K_i΄_ (3.8 and 4.6), which were determined from the slope of the secondary plots and Y-intercept of the LB plot against the concentrations of **3c**. The smaller K_i_ stipulated stronger inhibition of the enzyme which further indicated the possibility of an allosteric binding site in the enzyme where **3c** can bind. One way to confirm this would be a single crystal X-ray analysis of **3c**-α-glucosidase co-crystal. This is ongoing in our lab.

### *In vivo* studies

Male albino Wistar rats weighing between 200–240 gm each were used for the present investigation. The animals were categorized into five groups (I-V) containing three animals each (n = 3). Group-1 is the non-diabetic control (only drug vector), group-II is the positive test control (Streptozotocin [STZ] induced), group-III is the diabetes induced group with low dose of **3c** (3.6 mg/kg of the body weight), group-IV is also a diabetes induced group administered with **3c** at higher dose (7.2 mg/kg of body weight) and finally Acarbose (a standard drug) administered (10 mg/kg of body weight) diabetes induced group V. Each animals were housed in a separate cage at a temperature of 24 ± 3 °C, with relative humidity of 65 ± 5% and a 12 h dark-light cycle.

**Figure 6:**
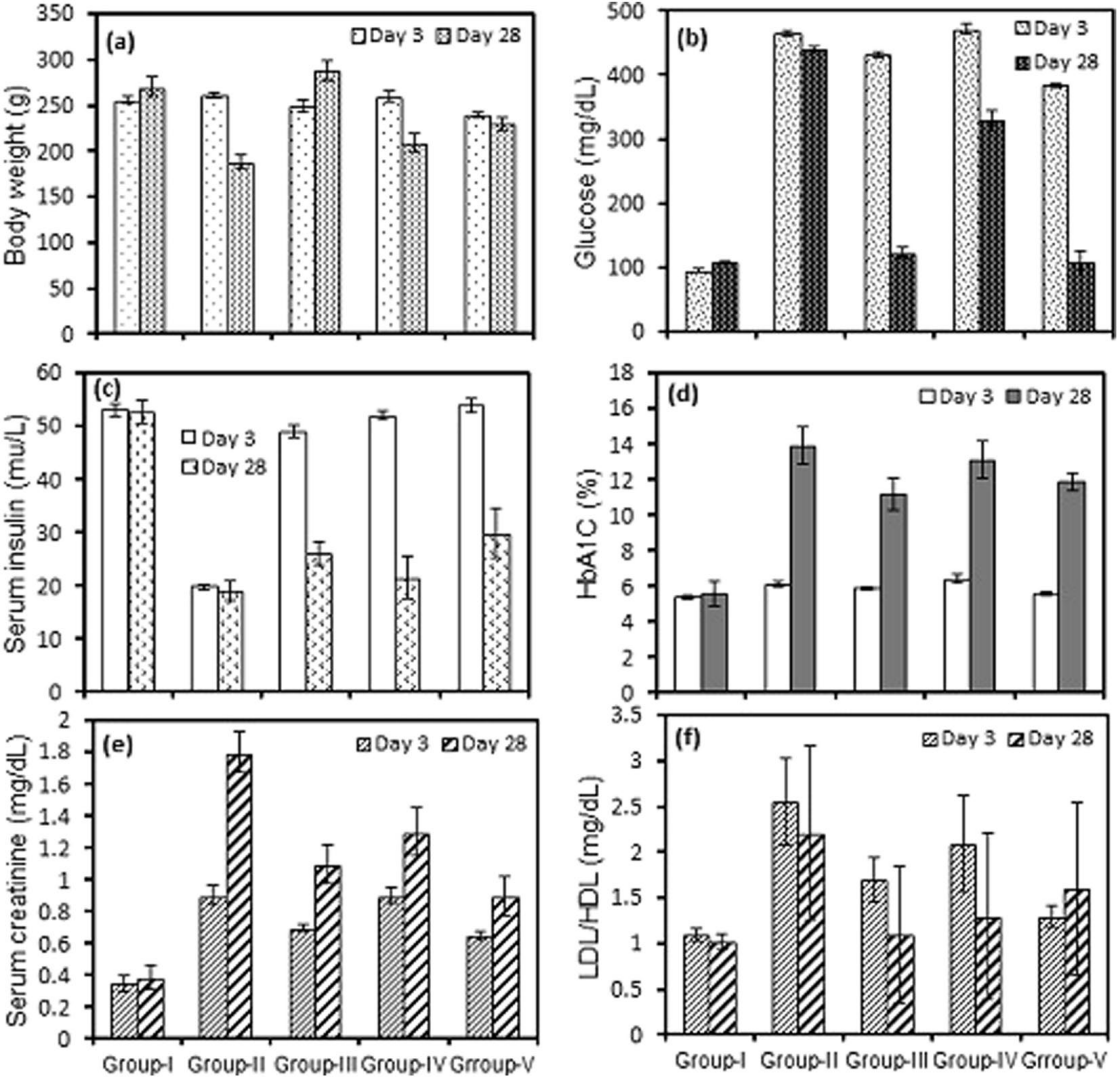
Effect of oral administration of **3c** at doses of 3.6 (group-III) and 7.2 mgkg^−1^ body wt. (group-IV) on (**a**) animal body weight; (**b**) serum glucose; (**c**) serum insulin; (**d**) HbA1C; (**e**) serum creatinine; (**f**) LDL/HDL ratio. Acarbose was administered at dose of 10 mgkg^−1^ body wt. for treating diabetes positive control rats (group-V). Each column represents mean ± SD for three rats (n = 3). Streptozotocin was used for inducing diabetes. The level of significance for the investigation was in between (P = 0.05-P = 0.001).

**Figure 7:**
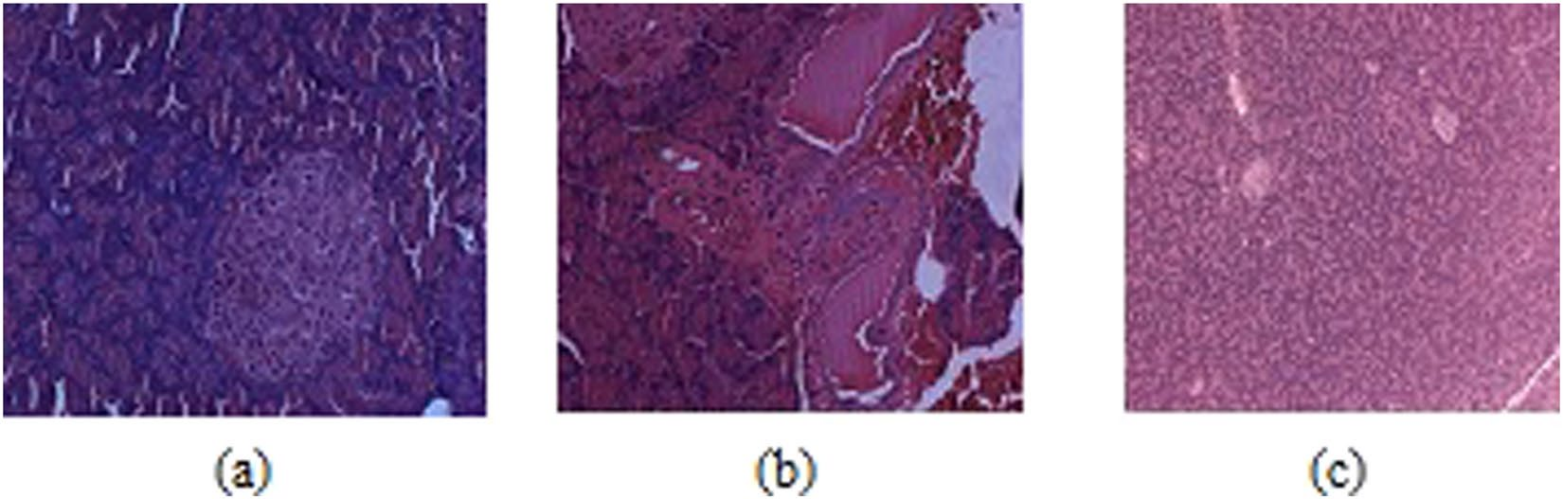
Histology of pancreas (**a**) normal case; (**b**) diabetic positive control, and (**c**) test compound high-dose treated.

**Figure 8:**
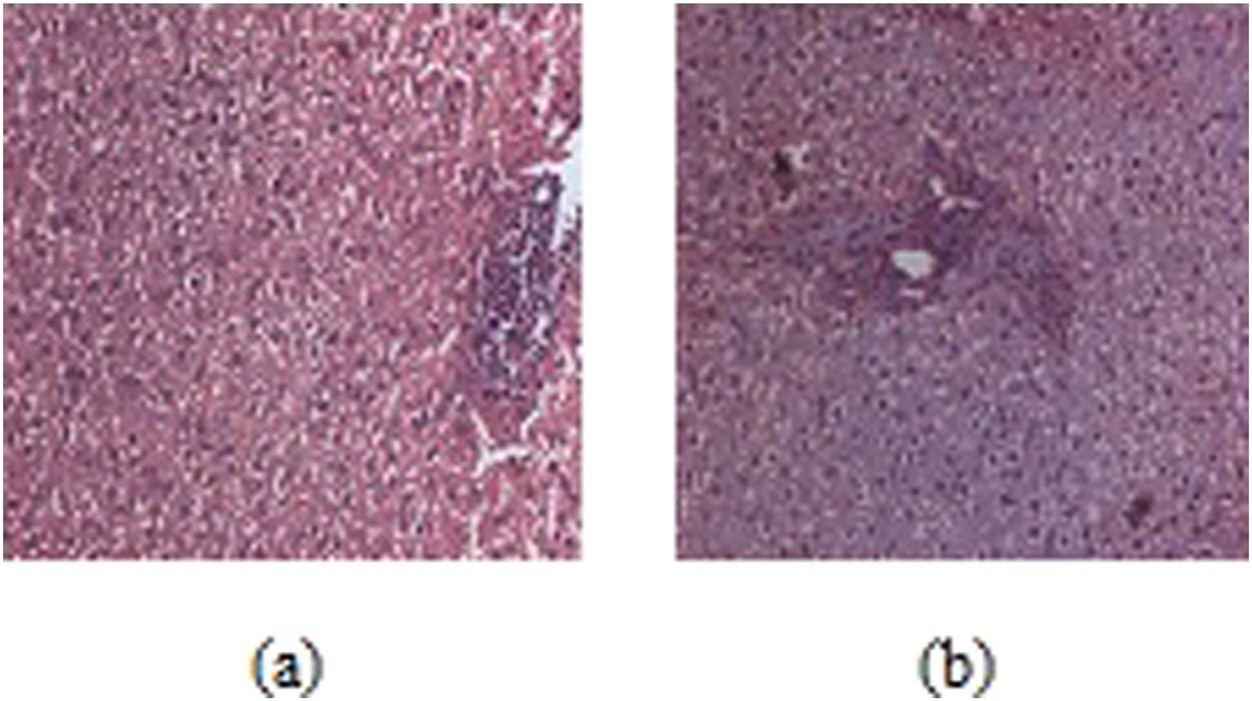
Histology of liver (**a**) diabetic positive control; (**b**) test compound high-dose treated.

The serum glucose level was measured in both the normal and diabetic rats on 3^rd^ and 28^th^ day, respectively, following the administration of **3c** and Acarbose, as positive standard. Three days after the STZ injection, the diabetic rats showed a significant (P < 0.001) increase in their fasting blood glucose (FBG) compared to the control group (Fig. 6b). There was a significant elevation in serum glucose, glycated haemoglobin (HbA1C) and serum creatinine while the serum insulin levels were significantly decreased in diabetic rats (Fig. 6b,c,d,e respectively). The administration of **3c** for about 4 weeks revealed a highly significant(P < 0.001) decrease in the FBG (fasting blood glucose) compared to the untreated diabetic rats. Moreover, lower dose of drug had better control over FBG in contrast to high-dose (Fig. 6b). This indicated that the drug is only an inhibitor of α-glucosidase at intestinal microvilli and perhaps at higher dosage of the drug was inhibiting α-glucosidase activity at an increased level and eventually decreasing the levels of blood glucose which resulted in the activation of hyperglycemic hormones for the maintenance of glucose levels^30,31^. Insulin levels dropped sharply after the administration of Streptozotocin, however, moderate improvement in the level of insulin was observed with **3c** after the treatment period (Fig. 6c). Further, the histological profile revealed atrophy of islet cells of pancreas in diabetic rats (Fig. 7a–c) and these animals exhibited moderate periportal inflammation in liver while mild inflammation was observed with rats treated with **3c** (Fig. 8a,b). As the blood glucose levels were controlled in response to **3c**, there could have been an opportunity for the pancreatic cells to repair in about 28 days which could lead to the improvement in insulin levels (Fig. 6c). The same was depicted in the histological changes associated with pancreatic cells i.e., shrinkage of β-cells in diabetic control while improved β -cell population in the presence of **3c** (Fig. 7a–c). Interestingly, insulin levels had not reached that of control values even after 28 days which might be due to the accumulation of toxic intermediates^32,33^.

The HbA1c increased significantly in diabetic rats, but, the response to the drug was not enough to make a change in their levels in comparison to that of nondiabetic rats (Fig. 6d) (at the end of the test period). Generally, the glycated haemoglobinis degraded over a period of three months, but, the change in HbA1c (from 11.9 → 13.9%) (P < 0.05) might beattributed to the formation of certain new population of RBC which were getting glycated at the lowering levels of glucose^34,35^. Elevation in the values of LDL to HDL ratio was observed with diabetes for about 28 days,but, the increase of this ratio was moderately high with low dosage of drug and it was slightly higher in case of high-dose which was controlled by the levels of secreted insulin (Fig. 6f). The insulin resistance observed after induction of diabetic condition led to the decrease in uptake of glucose which was excreted in the urine. This could result in increased breakdown of fat deposits and eventual increase of triglycerides, cholesterol and its esters^36,37^. Additionally, there could be decreased synthesis of apoprotein part of lipoprotein which could have the increased levels of VLDL compared to HDL^32^.

**Figure 9:**
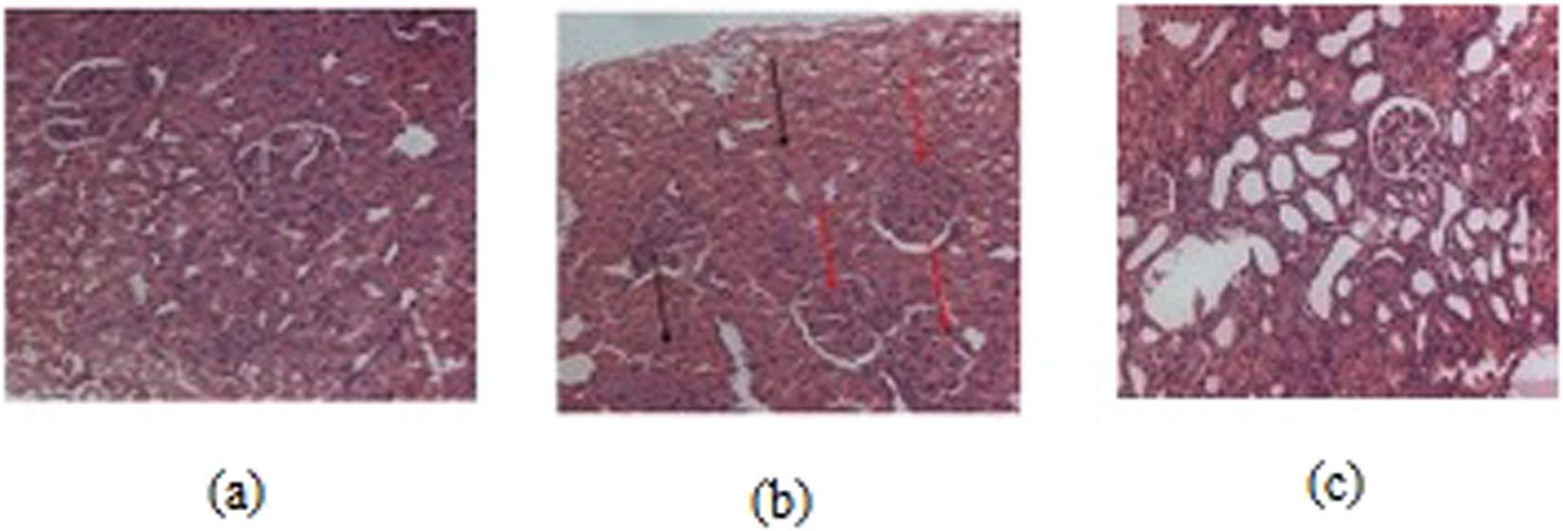
Histology of kidney (**a**) diabetic positive control; (**b**) test compound high-dose treated.

The serum creatinine clearence is based on the severity of diabetic condition^38,39^. Streptozotocin caused damage to nephrons and in turn facilitated serum clearance of creatinine from the destruction of muscle mass in severe diabetes with insulin resistance^40,41^. In the present investigation, there was an augmented levels of serum creatinine in the diabetic condition and moderately high levels of clearance with test animals (Fig. 6e). The change in the volume of skeletal muscle (Fig. 6a) and its concomitant effect on causing insulin resistance was also reflected in the serum creatinine levels. The condition was severe with interstitial inflammation and nephritis observed with diabetic control (Fig. 9a–c).

## Conclusion

Herein we have reported the discovery of a library of α-aryl ketones, as novel inhibitors of α-glucosidase (yeast). The design of these molecules included scaffold hopping and bioisosteric modification of lesser known natural product inhibitors of α-glucosidase i.e. **1** and **2**. The combinatorial strategy of compound generation involving a “shot-gun” synthesis of intermediates afforded a fifteen membered focused library (**3a**-**o**). *In vitro* screening of these molecules against α-glucosidase (yeast), α-fucosidase (bovine kidney) and α-mannosidase (jack bean) exhibited selective low micromolar inhibition of our compounds against α-glucosidase. The most active compound **3c** inhibited with an IC_50_ of 4.5 μM (acarbose, a standard drug inhibited α-glucosdase with an IC_50_ of 9.95 μM). Reaction kinetics study of **3c**, recommended it to be a mixed inhibitor which further indicated an allosteric binding of **3c** against α-glucosidase. *In vivo* study of **3c**, against male albino Wistar rats revealed that a lower dosage of 3.6 mg/kg of **3c** had better control over FBG (300 mg/dl → 120 mg/dl) in comparison to higher dosage (7.6 mg/kg), with minimal toxicity. This further suggested that **3c** may inhibit α-glucosidase at intestinal micro villi. Finally **3c** was docked against a homology model of α-glucosidase, to understand the putative binding sites. This proof of concept study provided fundamental information to begin an elaborate structure activity relationship campaign to identify a more potent α-arylketone as α-glucosidase inhibitors which eventually can be transformed into a preclinical candidate against type II diabetes.

## Materials and Methods

### Chemistry

Commercially available reagents and solvents were used without further purification. All reactions were monitored by thin-layer chromatography (TLC) using TLC plates (60 F254). The yields are not optimized. The purity of all compounds was over 95% as analyzed with an Agilent ZORBAX Eclipse Plus C18 (4.6 mm × 50 mm, 3.5 mm) reverse phase column, detecting with UV absorbance (254 nm). Purification of reaction products was carried out by flash chromatography using an InterchimpuriFlash®450 purification system with Interchim silica gel columns. ^1^H NMR and ^13^C NMR spectra were recorded with tetramethylsilane (TMS) as internal standard at ambient temperature unless otherwise indicated Bruker 400 MHz for ^1^H NMR and 100 MHz for ^13^C NMR. Chemical shifts are reported in parts per million (ppm) and coupling constants are reported as Hertz (Hz). Splitting patterns are designated as singlet (s), broad singlet (bs), doublet (d), triplet (t). Splitting patterns that could not be interpreted or easily visualized are designated as multiple (m). The Mass Spectrometry analysis was done on the 6540 UHD Accurate-Mass QTOF LC/MS system (Agilent Technologies) equipped with Agilent 1290 LC system obtained by the Dept. of Chemistry, School of Natural Sciences, Shiv Nadar University, Uttar Pradesh 201314, India.

### Enzymatic assays

The inhibitory effect of the synthesized compounds on α-glucosidase (yeast origin), α-fucosidase (bovine kidney origin) and α-mannosidase (jack bean origin) inhibitory activity was determined in 96-well plates employing the substrate PNPG and 4-nitrophenyl α-D-glucopyranoside according to the procedure previously reported by Ferreres *et al*.^42^. Prior to use, all test compounds were solubilised in solvent, dimethylsulfoxide (DMSO), and then further diluted in DMSO to acquire the desired final maximum test concentration. Briefly, each well in 96-well plates contained 100 µL of 2 mM 4-nitrophenyl α-D-glucopyranoside (PNP-G) in 10 mM potassium phosphate buffer (pH 7.2) and different test concentrations (2–10 µM). The reaction was initiated by the addition of 5 µL of the enzyme solution (0.1 IU per well). α-glucosidase (yeast), α-fucosidase (bovine kidney) and α-mannosidase (jack bean) were purchased from Sigma Aldrich, Bangaluru). The plates were incubated at 37 °C for 10 min. The absorbance was measured spectrophotometrically at 430 nm (Spectra Max M5e micro plate reader). The increase in absorbance (ΔA) was compared with that of the control (buffer instead of test compound) to compute the inhibitory concentrations (IC_50_) which was determined from two independent assays, performed in duplicate. Acarbose, an illustrious inhibitor of α-glucosidase was employed as positive control.Inhibition (%) = (**Δ**A_control_ − **Δ**As_ample_/**Δ**A_control_ × 100).The concentration of compound required to obtain 50% inhibition ofα-glucosidase activity under the assay conditions was defined as the IC_50_ value.

### Inhibition kinetics

The kinetic mode of inhibition of the highest active compounds against α-glucosidase was determined by preparing a series of test solutions in which the concentration of the respective substrate was varied in the presence of different concentrations of the inhibitors (10–50 μM). The mode of inhibition (i.e. competitive, non-competitive, uncompetitive or mixed-type) of the screened compounds was determined on the basis of the inhibitory effects on K_m_ (dissociation constant) and V_max_ (maximum reaction velocity) of the enzyme^36^. These studies were computed using the primary (Lineweaver–Burk plot) plots, which are the double reciprocal plots of enzyme reaction velocities (1/V) versus substrate concentrations (1/[S]). Analysis of the same data by secondary plots of slope versus [inhibitor] and Y-intercept versus [inhibitor] were also performed. The Lineweaver–Burk (LB) equation follows as:

$$V=\frac{{V}_{max}[S]}{Km[1+\frac{[I]0}{{K}_{i}}]+[S][1+\frac{[I]0}{{K}_{i}^{^{\prime} }}]}$$

### Animal

Male albino Wistar rats weighing between 200–240 g each were used for the present investigation. The animals were categorized into five groups (I-V) of three each (n = 3), while each one was housed in a separate cage at a temperature of 24 ± 3 °C, with relative humidity of 65 ± 5% and a 12 h dark-light cycle. They were housed and handled in Biosafety Level 2 (BSL2) animal facilities at University College of Pharmaceutical Sciences, Kakatiya University, Warangal, Andhra Pradesh, India and were provided with a standard pellet diet of laboratory and water with continuous monitoring. Actual drug administration was begun after 72 h of diabetic induction by streptozotocin (STZ). The test drug acarbose, a known α-glucosidase inhibitor were administered orally every day for about 28 days soon after the food was completed by the animal. All protocols were reviewed prior to the start of the experiment by an independent ethical review board at University College of Pharmaceutical Sciences, Kakatiya University and approved to be in accordance with the license for animal experiments issued by The Animal Experiments Inspectorate (License no. 1820/GO/Re/S/CPCSEA).

### Induction of Type 2 diabetes

Diabetes was induced postprandial in the rats (Group II-V)bya single intraperitoneal (I.P) injection of freshly prepared Streptozotocin(60 mgkg^−1^ body wt.) in 0.1 M sodium citrate buffer of pH 4.5. Non-diabetic control rats (group-I) were received an I.P injection of buffer alone. Diabetes was confirmed after 72 h of STZ induction by assaying the blood glucose levels. The rats having blood glucose higher than 11 mmol.L^−1^ were considered to be diabetic and selected for further study.

### Determination of biochemical parameters and weight of the animal

For the determination of biochemical parameters, the animals were kept under fasting overnight and peripheral blood was drawn for the assays. The tests were carried out using commercial kits and following the manufacturer’s instructions.At the end of test period of 28 days, the animals under fasting conditions were anesthetized and sacrificed. Blood and tissues were stored for further biochemical and histological studies. Each animal in every group was tested for body weight, blood glucose, insulin, creatinine, Hb1Ac, lipid profiles.

### Statistical analysis

The results were expressed as mean ± standard deviation (M ± SD) for three rats in each group. The differences between treated and control groups were assessed by one way analysis of variance (ANOVA) using MINITAB (Version. 14). The values of p < 0.05 were considered to show the statistical significance.

### Molecular docking

#### Homology modeling

The sequence of alpha-glucosidase was downloaded from Uniprot (ID: P10253). Blastp against protein data bank database was performed in order to identify the template for sequence alignment. Human Maltase-Glucoamylase (PDB ID: 2QLY, 3L4T) were showing good similarity to our query sequence. Residues starting from 89 are aligning to these PDB and showing 44% identity. So we select these two protein structure to model alpha-glucosidase using homology modelling. The homology model of alpha-glucosidase was built using Modellerv9.14. Three models were generated using Modeller v9.14 and model having best DOPE score is selected for further refinement. Modelled structure consist of certain loops structure that are further refined using ModLoop server. The energy of the refined model was minimized using maestrov10 using Force Field OPLS2005. The stereochemical quality of this model is validated by Ramachanadranplot. More than 99.3% residues are in favored and allowed regions and only 0.7% (6 residues) are in outlier region.

#### Preparation of modelled alpha glucosidase protein structure

Protein preparation was performed using protein preparation wizard of maestro v10 in which assign bond orders, addition of hydrogens, create di-sulfide bonds were done. After that H-bond assignment were optimize using PROPKA at pH 7.0. Finally the energy of the protein were minimized using OPLS2005 force field.

#### Ligand preparation

The 2D structures of the compounds were build using MOE-Builder tool. After this various 3D conformation was search using conformational search tool in MOE. The conformation with least energy was selected for docking studies.

#### Docking

For docking studies both the modelled structure and template structure (PDB ID: 3L4T) were aligned and the centroid coordinates of the complex ligands in the crystal structure (PDB ID: 3L4T) were used as the center of the docking site. A grid of 60 × 60 × 60 with 0.375 Ǻ were constructed around the docking area using Autogrid v 4.2 software. Docking analysis were done using Autodock v 4.2 software in which top 10 docked conformation were taken using Genetic Algorithm. Each docking calculation consist of 25 × 10^6^ energy evaluations with 250 population size. A mutation rate of 0.02 and a crossover rate of 0.8 were used to generate new docking trials.
